# Design of a wasp-inspired biopsy needle capable of self-propulsion and friction-based tissue transport

**DOI:** 10.3389/fbioe.2024.1497221

**Published:** 2025-01-06

**Authors:** Jette Bloemberg, Suzanne van Wees, Vera G. Kortman, Aimée Sakes

**Affiliations:** ^1^ Department of BioMechanical Engineering, Bio-Inspired Technology Group, Faculty of Mechanical Engineering, Delft University of Technology, Delft, Netherlands; ^2^ Department of Biomedical Engineering, Faculty of Science and Engineering, University of Groningen, Groningen, Netherlands

**Keywords:** bio-inspired design, biomimetics, medical device design, minimally invasive surgery, ovipositor, pancreatic biopsy

## Abstract

Percutaneous pancreatic core biopsy is conclusive but challenging due to large-diameter needles, while smaller-diameter needles used in aspiration methods suffer from buckling and clogging. Inspired by the ovipositor of parasitic wasps, which resists buckling through self-propulsion and prevents clogging via friction-based transport, research has led to the integration of these functionalities into multi-segment needle designs or tissue transport system designs. This study aimed to combine these wasp-inspired functionalities into a single biopsy needle by changing the interconnection of the needle segments. The resulting biopsy needle features six parallel needle segments interconnected by a ring passing through slots along the length of the needle segments, enabling a wasp-inspired reciprocating motion. Actuation employs a cam and follower mechanism for controlled translation of the segments. The needle prototype, constructed from nitinol rods and stainless steel rings, measures 3 mm in outer diameter and 1 mm in inner diameter. Testing in gelatin phantoms demonstrated efficient gelatin core transport (up to 69.9% ± 9.1% transport efficiency) and self-propulsion (0.842 ± 0.042 slip ratio). Future iterations should aim to reduce the outer diameter while maintaining tissue yield. The design offers a promising new avenue for wasp-inspired medical tools, potentially enhancing early pancreatic cancer detection, thus reducing healthcare costs and patient complications.

## 1 Introduction

### 1.1 Pancreatic tissue sampling

Pancreatic cancer is increasingly common and highly fatal, necessitating effective detection methods (Partyka et al., 2023; Rahib et al., 2014; Sarantis et al., 2020). Diagnosis relies heavily on histological and cytological analysis of masses or lesions (Tyng et al., 2015; Mittal et al., 2023; Balen et al., 1994), often through percutaneous biopsy procedures (Yamao et al., 2005; Crowe et al., 2006; Voss et al., 2000).

Ideally, a biopsy needle should be thin to minimize complications like bleeding and patient discomfort while providing continuous sampling of intact tissue. Fine Needle Aspiration Biopsy (FNAB) and Core Biopsy (CB) are the primary percutaneous methods, with FNAB using thin needles (22–25 G) to retrieve cytological and fluid samples using aspiration and CB employing thicker needles (19–13.5 G) to obtain an intact cylindrical tissue sample, called a core, via a discrete cutting action (May et al., 2018).

Despite their slenderness and ability for continuous sampling, FNAB needles for pancreatic biopsies have been associated with a higher incidence of false negatives compared to CB needles (Liu et al., 2023; Caymaz and Afandiyeva, 2023). Tikkakoski et al. (1992) reported a 13% false negative rate in ultrasound-guided fine-needle pancreatic biopsy. This high rate may stem from needle deflection due to buckling, which can cause the needle to miss the target area, a known issue in breast biopsies (Sabel, 2009). Additionally, aspiration-based methods can result in device clogging, making them unreliable for obtaining intact tissue samples needed for histological evaluation (Madjidyar et al., 2019). The push towards smaller, less invasive tools highlights the need for improved biopsy methods that balance needle size with tissue sampling efficacy.

### 1.2 Bio-inspiration: ovipositor-based tools for MIS

In an attempt to overcome clogging problems in aspiration-based devices, devices that employ alternative mechanisms for tissue transport have been developed. For instance, Kortman et al. (2023) developed a cable-actuated conveying mechanism for tissue transport that uses the friction between continuously rotating conveying cables and tissue. To overcome buckling problems in thin needles, extensive research is ongoing into lowering the needle’s penetration load by optimizing the needle tip design, introducing needle vibrations, increasing the insertion speed, and increasing skin tension factors (Hirsch et al., 2012; Lenau et al., 2017; Irwin et al., 2021). However, needle designs that can overcome both clogging and buckling problems have yet to be shown.

**FIGURE 1:**
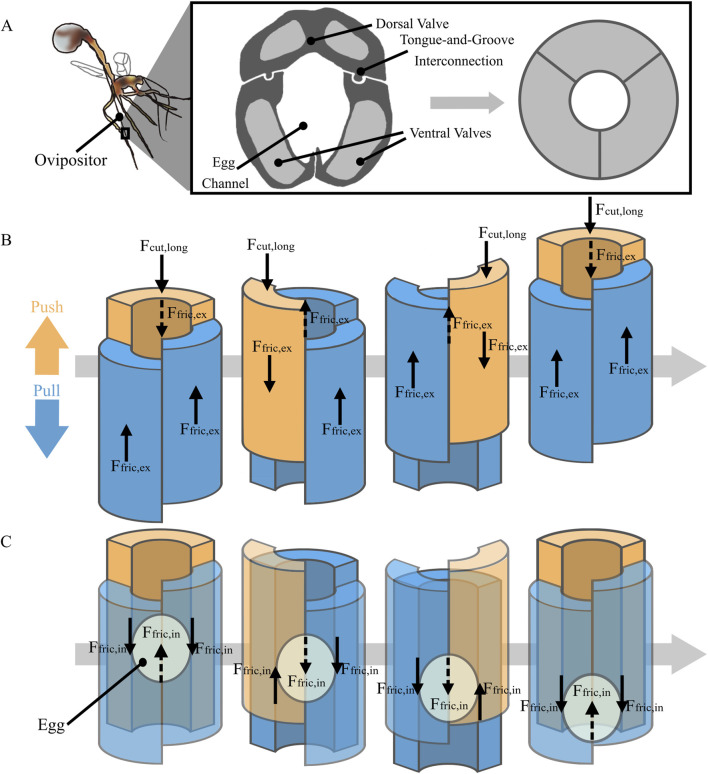
Schematic representation of the wasp ovipositor structure and movement. **(A)** Cross-section of the ovipositor of the parasitic wasp, showing three valves, the egg channel, and the tongue-and-groove interconnection, with a simplified schematic of the valves. **(B)** Ovipositor’s reciprocating push-pull cycle for self-propulsion. In this cycle, one valve (orange) advances, encountering longitudinal cutting $\left(F_{\text{cut,long}}\right)$ and external friction force $\left(F_{\text{fric,ex}}\right)$ , whilst the other two valves (blue) retract but remain stationary with respect to the surrounding substrate due to external friction force $\left(F_{\text{fric,ex}}\right)$ applied on them by the surrounding substrate. **(C)** Ovipositor’s push-pull cycle for egg transport. In this cycle, one valve (orange) advances, creating internal friction $\left(F_{\text{fric,in}}\right)$ with the egg, while the other two (blue) retract, generating greater internal friction force ( $\left(F_{\text{fric,in}}\right)$ , moving the egg downwards. The dotted lines indicate forces applied by the back valve.

In nature, the ovipositor of parasitic wasps, used for depositing eggs deep into substrates (King and Vincent, 1995; Sakes et al., 2016), is a remarkable biological structure resembling biopsy needles, as both are long, thin structures designed to penetrate a substrate while simultaneously transporting something (either an egg or tissue) through the lumen. Despite usually having a diameter of only 0.2–0.3 mm, some ovipositors can penetrate up to 20 mm into trees to lay their eggs (Cerkvenik et al., 2017; Quicke and Fitton, 1995). The structure of the ovipositor is different from traditional biopsy needles as it consists of three longitudinally connected valves, one dorsal and two ventral. These valves surround the channel that the egg passes through, as can be seen in Figure 1A. The valves feature a tongue-and-groove mechanism called the olistheter, facilitating a longitudinal sliding motion of the individual valves (Quicke et al., 1994). During drilling into substrates, the olistheter enables the valves to translate in a continuous reciprocating manner, with one valve advancing (i.e., push) whilst the other two valves retract (i.e., pull) in a continuous cycle (Cerkvenik et al., 2017; van Meer et al., 2020). It has been hypothesized that this reciprocating movement gives rise to two functionalities: 1) self-propulsion and 2) friction-based egg transport. These functionalities have inspired the designs of two distinct types of medical devices: self-propelling needles and friction-based tissue transporters.

#### 1.2.1 Self-propelling needles

Self-propelling needles are inspired by the ovipositor’s ability to propel through substrates without buckling. The self-propelling motion is achieved because the longitudinal forces on the advancing valve are counterbalanced by the longitudinal forces on the retracting valves (Scali, 2020; Leibinger et al., 2016; Sakes et al., 2016). The advancing valve is pushed into the substrate and experiences a cutting force from substrate deformation in front of the valve and an external friction force caused by contact with the surrounding substrate (Okamura et al., 2004; Fung-A-Jou et al., 2023; Yang et al., 2018). Similarly, the retracting valves experience a friction force in the opposite direction as they are retracted. Although a pulling force on the retracting valves would cause them to retract in free air, we assume that within a substrate, external friction forces prevent relative sliding between the retracting valves and the surrounding substrate, causing the ovipositor to propagate into the substrate over one cycle. Figure 1B shows a schematic representation of the ovipositor and relevant forces during self-propulsion. The self-propelling motion can result in the needle moving forward with a zero net push force on the needle, making it less prone to buckling as it propagates through a substrate. To achieve a net zero external push force, the forces on the valves or needle segments should be in balance, as indicated in Equation 1.

$$-\sum \left(F_{\text{fric,ex,ad}}+F_{\text{cut,long}}\right)=\sum F_{\text{fric,ex,re}}$$ (1)

Where $F_{\text{fric,ex,ad}}$ and $F_{\text{fric,ex,re}}$ denote the friction forces on the external surfaces of the advancing and retracting valves interacting with the substrate in Newtons, respectively. $F_{\text{cut,long}}$ represents the cutting force on the advancing valve in the longitudinal direction in Newtons. Inertial forces on the valves are considered negligible due to their minimal mass, and inter-valve friction forces are neglected.

Within the medical field, multiple self-propelling needles inspired by the ovipositor have been developed. Studies by Oldfield et al. (2015), Frasson et al. (2010), and Leibinger et al. (2016) demonstrated that multi-segment needles with reciprocating motion can self-propel, which reduces tissue displacement and tissue damage around the needle compared to traditional needles, which are pushed into the body. Due to challenges in scaling down the tongue-and-groove interconnection method of the ovipositor while integrating flexibility, Scali et al. (2019) and Bloemberg et al. (2022) used parallel-oriented nitinol rods of equal, circular cross-section to mimic the valves. A heat shrink tube was used to bundle the rods, resulting in sub-millimeter diameter needles in both designs. Self-propulsion of a wasp-inspired needle is accomplished by a set of parallel needle segments that can both advance or retract with respect to one another. The advancing needle segments experience both a cutting force due to the tissue’s plastic deformation and stiffness and a friction force along the length of the needle in contact with the surrounding tissue (Ng et al., 2013). The retracting needle segments, however, only experience a friction force. The needle self-propels through the tissue if the friction generated by the retracting needle segments overcomes the friction and cutting forces of the advancing needle segments. This can be achieved by keeping the number of advancing needle segments lower than the number of retracting needle segments (as in Equation 1). Therefore, at least three needle segments are required for the self-propulsion principle.

#### 1.2.2 Tissue-transportation devices

Besides facilitating self-propulsion, the reciprocating translation of the ovipositor valves has also been hypothesized to facilitate egg transport from the body of the wasps to the ovipositor tip (Austin and Browning, 1981). Friction forces between the egg and the internal surface of the ovipositor valves $F_{\text{fric,in}}$ are thought to govern this transport as the ovipositor valves move in their reciprocating manner. The net longitudinal force on the egg $F_{\text{egg,long}}$ , measured in Newtons, to move the egg through the ovipositor equals the sum of internal friction forces $\left(F_{\text{fric,in}}\right)$ in Newtons (Equation 2).

$$F_{\text{egg,long}}=\sum F_{\text{fric,in}}$$ (2)

For successful egg deposition, the frictional force between the advancing valves and the egg must exceed that of the retracting valve and the egg (Equation 3). In this case, the egg will move with the advancing valves, towards the tip. Conversely, for movement in the opposite direction, in which the egg moves from the tip of the ovipositor towards the base, like a tissue sample being retrieved, this condition should be inverted (Equation 4). In Figure 1C., a schematic representation of the ovipositor and the relevant forces during egg transport from the tip towards the base of the ovipositor are provided.

$$\sum F_{\text{fric,in,ad}}>\sum F_{\text{fric,in,re}}$$ (3)

$$\sum F_{\text{fric,in,ad}}<\sum F_{\text{fric,in,re}}$$ (4)

Here, $F_{\text{fric,in,ad}}$ and $F_{\text{fric,in,re}}$ are the friction forces inside the ovipositor, between the egg and the advancing and retracting valves, respectively. All forces are measured in Newtons. Gravity’s influence on egg transport is considered negligible due to the egg’s small mass.

Multiple friction-based tissue-transport designs have been inspired by the egg movement through the ovipositor. These designs are used to transport tissue samples from the distal end inside the body to the local base outside the body for extraction purposes. Sakes et al. (2020) created a morcellator design ( $\emptyset_{\text{outer}}$ : 7 mm, $\emptyset_{\text{inner}}$ : 4.5 mm) resembling the ovipositor by mimicking its segments as blades. Instead of a tongue-and-groove mechanism, the interconnection was facilitated using the stiffness of the blades and an external brass tube to prevent outward motion. de Kater et al. (2021) replaced the blades with magnetic galvanized steel cables ( $\emptyset_{\text{outer}}$ : 10 mm, $\emptyset_{\text{inner}}$ : 5 mm) to integrate flexibility into the system. To maintain an open central lumen for tissue transport, ring magnets were placed around the cables, forming a tubular structure with a lumen, without internal protrusions.

In order to initiate friction-based tissue transport, the tissue to be transported needs to experience a resultant friction force with the surrounding parallel cables in the desired transport direction. The resultant friction force comprises advancing frictional components and retracting frictional components corresponding to the motion of the cables. If we assume that the gravity of the tissue that needs to be transported can be neglected, tissue transportation from the distal end to the local base can be achieved if the sum of the friction between the retracting cables and the tissue exceeds the sum of the friction between the advancing cables and the tissue. This can be achieved by keeping the number of advancing cables lower than the number of retracting cables (as in Equation 4). Therefore, at least three surrounding cables are required for the friction-based tissue transport.

### 1.3 Combining self-propulsion and tissue transport into a wasp-inspired biopsy needle

**FIGURE 2:**
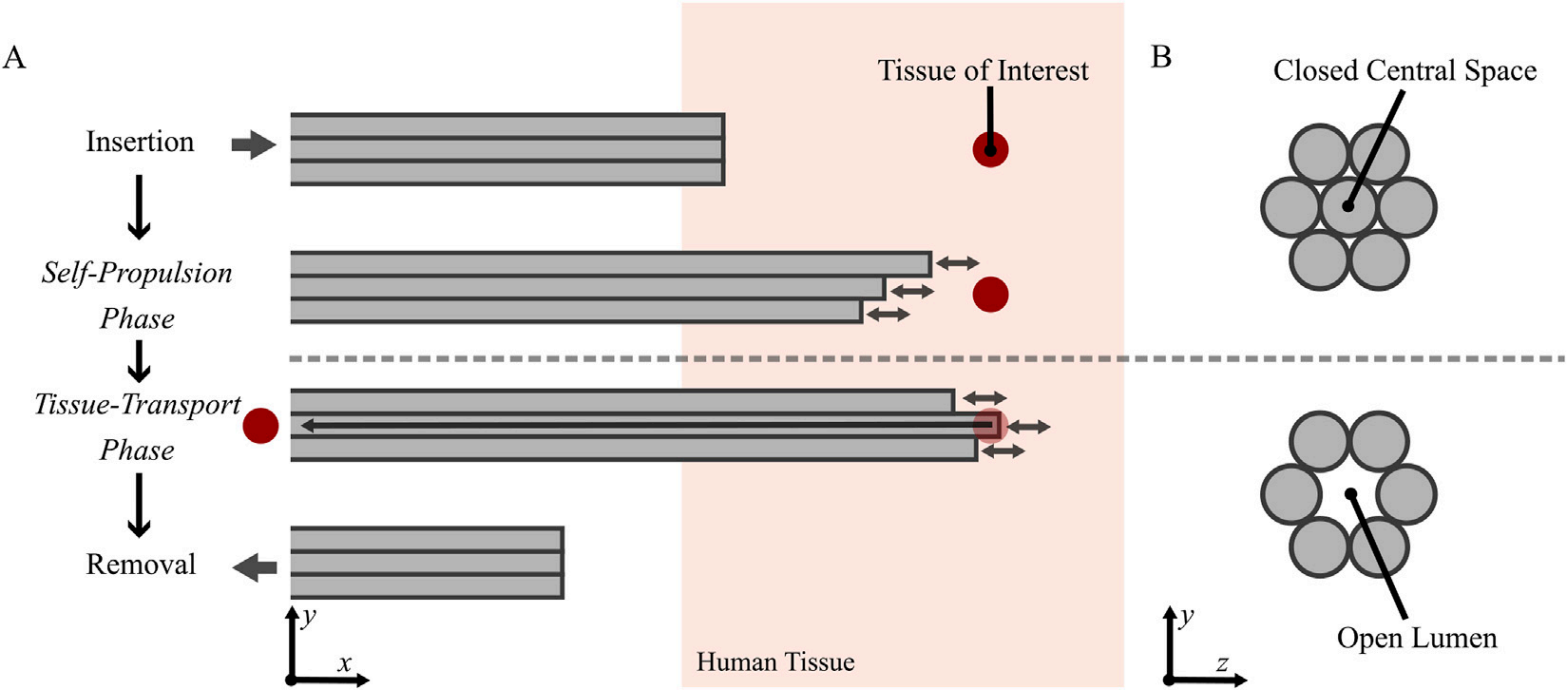
**(A)** Schematic illustration of functioning of a wasp-inspired biopsy needle in distinct phases: Insertion into tissue, self-propulsion to the tissue of interest, tissue transport through the lumen (and removal of the tissue sample for examination), and removal of the biopsy needle from the tissue. **(B)** Desired cross-sections of the wasp-inspired biopsy needle during the different phases: During the Insertion and the Self-Propulsion Phase, the central space remains closed to prevent the needle from inadvertently resecting the tissue. In the Tissue-Transport Phase, a lumen is required to enable tissue transport through the needle.

Integrating both self-propulsion and friction-based tissue transport into a single slender design, inspired by the ovipositor, could offer a novel alternative to current biopsy needles. This design could self-propel to a tissue of interest and use friction-based transport to move the tissue sample through the biopsy needle in two distinct phases: 1) the self-propulsion phase and 2) the tissue-transport phase. The phases and required needle cross-sections during the phases are illustrated in Figures 2A, 2B, respectively.

By combining both the self-propulsion and transport principles of the ovipositor of parasitic wasps, deflection and clogging issues of current thin biopsy needles are negated by relying on friction for both needle insertion and tissue transport. However, combining these functionalities is challenging. A biopsy needle with multiple parallel segments forming a lumen, similar to the ovipositor structure, depends on external friction between the external surface of the needle segments and surrounding tissue for self-propulsion, as well as on internal friction between the tissue sample and the internal surface of the segments for tissue transport. Any interconnecting structure must not interfere with both internal and external contact surfaces. As shown in previous designs, moving away from the ovipositor-inspired tongue-and-groove mechanism is necessary to miniaturize the needle dimensions (Scali et al., 2019; Bloemberg et al., 2022) and to introduce flexibility (de Kater et al., 2021). Former tissue-transport designs have maintained a lumen using external structures that interfere with external surface contact, whereas former self-propelling needles have no lumen to facilitate internal surface contact, highlighting the difficulty of integrating both wasp-inspired functionalities into a slender, flexible biopsy needle.

### 1.4 Goal of this study

This study aims to combine the two ovipositor-inspired functionalities, self-propulsion and friction-based tissue transport, into a single, slender biopsy needle design. This approach could offer a viable friction-based alternative to current biopsy needles, particularly for deep biopsies like pancreatic percutaneous biopsies that are currently limited by issues such as buckling and clogging in smaller, aspiration-based needles. Building on previous flexible needle and tissue-transport designs using parallel rods to mimic the valves of the ovipositor, this study will adapt the interconnection method of these needle segments and evaluate the potential of the integrated design as a biopsy needle.

## 2 Proposed biopsy needle design

### 2.1 Needle

**FIGURE 3:**
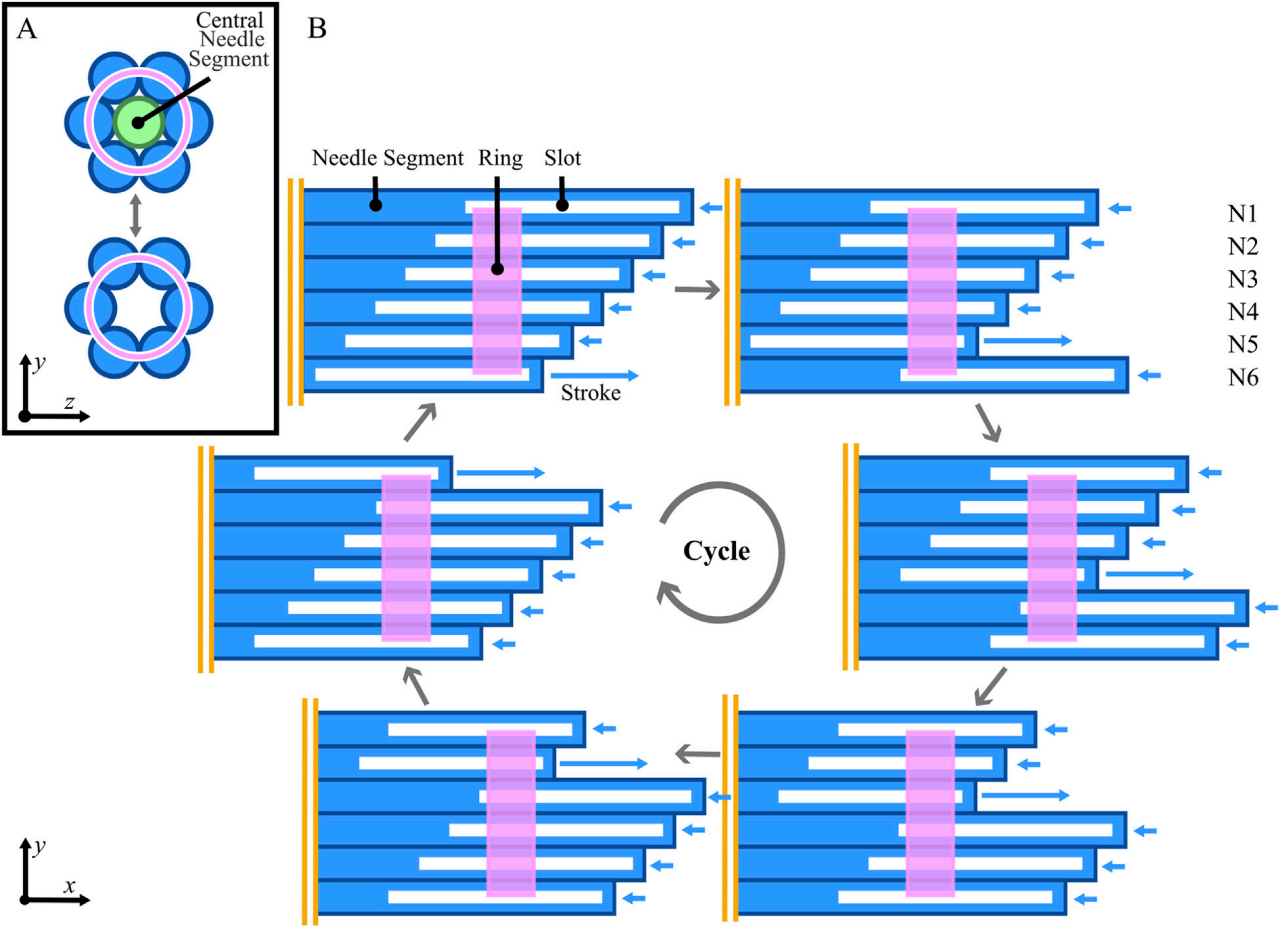
Working principle of the ring-through-slots interconnection of the biopsy needle. **(A)** the ring (pink) constrains radial translation and rotation of six needle segments (N1-N6, blue), maintaining a hexagonal configuration. This can be with or without a central needle segment to fill the lumen (light green) depending on the corresponding phase of the procedure. **(B)** 2D flattened representation of the longitudinal translation of the individual needle segments over one cycle using a 1:5 motion sequence. In this motion sequence, the needle segments are advanced one-by-one over one stroke distance (S) while retracting the other five segments by one-fifth of this stroke distance. The vertical parallel lines (orange) indicate that this is a visualization of the tip of the needle and the segments extend further on this side.

The proposed wasp-inspired biopsy needle design consists of six parallel needle segments that mimic the ovipositor valves. A total number of three outer needle segments would have been sufficient to ensure that the number of retracting needle segments exceeds the number of advancing needle segments. However, to enable a central lumen for tissue transport, we opted for six outer needle segments and a seventh central needle segment that can be removed before the tissue-transport phase. Each needle segment has an equal circular cross-section and a beveled tip. The six needle segments are held in a hexagonal arrangement by a ring passing through slots along the length of the segments, as shown in the cross-sectional view in Figure 3A. This ring-through-slot interconnection restricts the radial translation while allowing longitudinal translation of each needle segment for self-propulsion and tissue transport. The interconnection also prevents rotation of the needle segments around their longitudinal axis, maintaining the orientation of the beveled tips towards the center.

The individual longitudinal translation of the needle segments allows the biopsy needle to mimic the reciprocating movement of the ovipositor valves. Instead of using the 1:2 reciprocating movement sequence of the ovipositor, where one valve is advanced as two are retracted, a 1:5 motion sequence, as used in previous self-propelling designs (Bloemberg et al., 2022) and tissue-transport designs (Sakes et al., 2020; de Kater et al., 2021) is used. The six needle segments are longitudinally translated using this motion sequence over a cycle of six steps. During each step, one needle segment moves forward by a specified distance, called the stroke length (S), while the other five segments move backward by one-fifth of this stroke length ( $\frac{1}{5}$ S). After one cycle, the needle segments return to their starting positions, as illustrated in Figure 3B.

**FIGURE 4:**
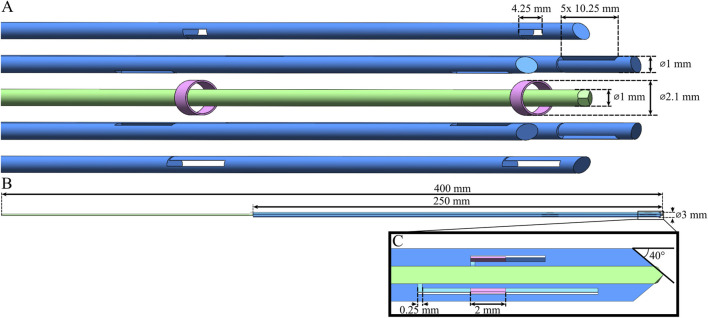
Conceptual design of the wasp-inspired biopsy needle. **(A)** Exploded view of the needle design, consisting of six needle segments with slots (blue), two interconnecting rings (pink), and one central needle segment (light green), indicating their respective diameters and slot sizes. **(B)** Assembly of the needle, indicating its total diameter and length of the needle segments with slots and the central needle segment. **(C)** Cross-section of the needle tip, indicating the bevel angle of the needle segments, the width of the L-shaped slot, and ring length.

The needle segments were designed to be 1 mm in diameter, allowing for the transport of a cylindrical tissue sample, or core, of 1 mm in diameter, which is a common diameter for biopsy samples (Rogowska, 2022). The total diameter of the biopsy needle was 3 mm. A trade-off existed in the number of slots introduced into the needle segments: introducing more slots along the length of the needle could potentially comprise the ability of each needle segment to resist the cutting forces, whilst this simultaneously is advantageous for maintaining the hexagonal arrangement of the entire needle. To balance this, we introduced two slots with a spacing of 100 mm. The first slot was placed near the base of the needle and the second slot was positioned 4 mm from the needle tip to prevent the needle segments from diverging at the tip during self-propulsion. To facilitate the assembly of the needle segments and rings, the slots were not designed to be rectangular but L-shaped to provide an entry point for the rings. Figure 4 shows the concept design and assembly of the needle.

The six needle segments enclose the tissue transportation lumen, which must be either filled or unfilled depending on the biopsy procedure phase (Figure 2B). During the self-propelling phase, the biopsy needle travels to the tissue of interest. The lumen should be filled to prevent a cylindrical piece of tissue from being cut out as the biopsy needle travels into the tissue. To achieve this, a seventh needle segment (indicated in green in Figure 4A) was introduced in the design to fill the lumen when needed. This central needle segment ideally participates in the reciprocating movement during the self-propelling phase and should be able to be removed during the tissue-transport phase, when a lumen is needed.

### 2.2 Actuation

**FIGURE 5:**
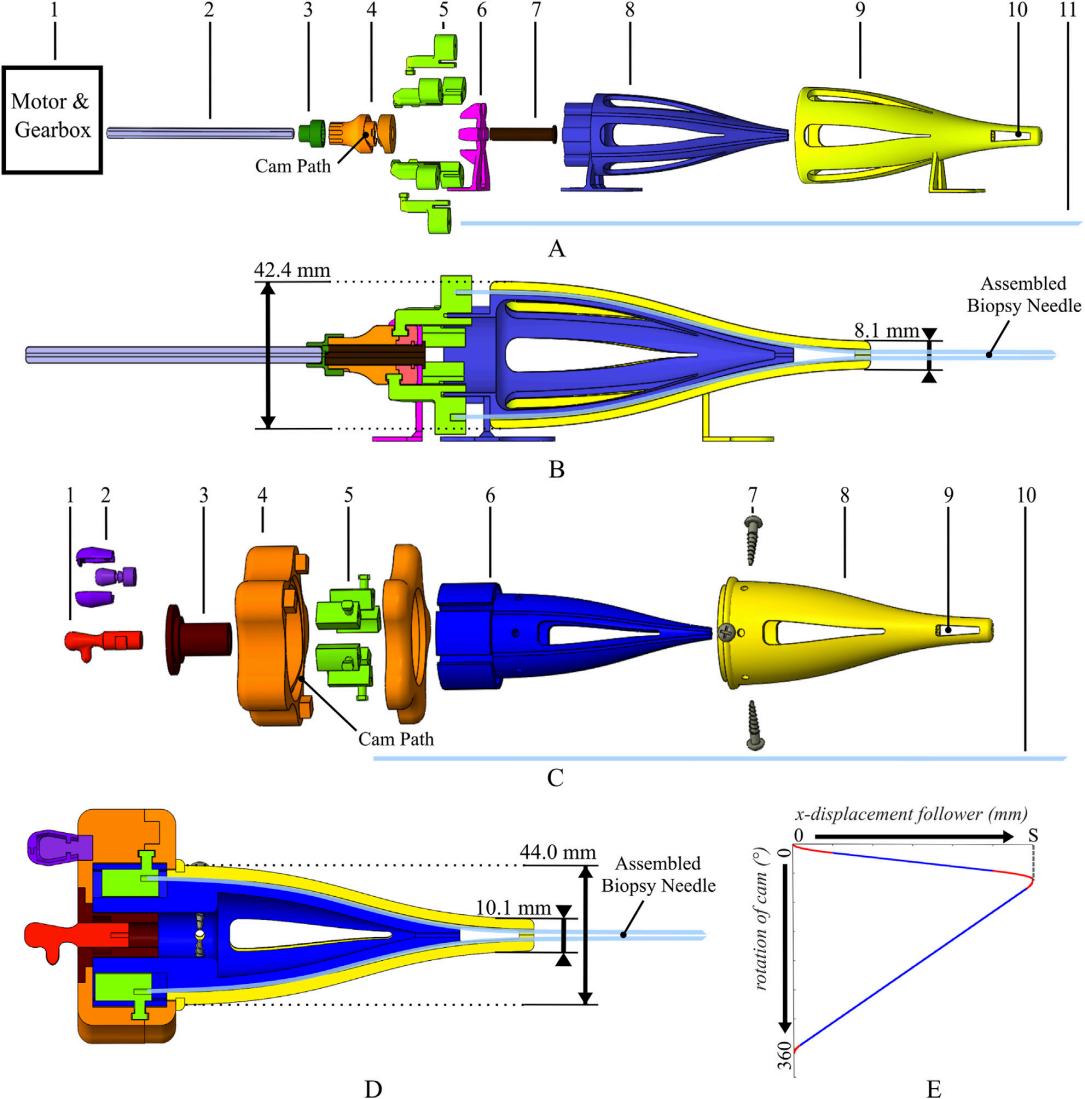
Exploded views and cross-sections of the motorized actuation unit **(A, B)** and manual actuation unit **(C, D)** for the wasp-inspired biopsy needle. The motorized actuation unit consists of a motor and gearbox (A.1), axle (A.2), cam connector piece (A.3), cam (bottom and top) (A.4), needle segment holders (followers) (A.5), supporting structures (A.6, A.7), inner and outer converging cone (A.8, A.9), outer cone opening (A.10) and needle segments (A.11). The manual actuation unit consists of a central needle segment connector (C.1), a spinner knob (C.2), supporting structure (C.3), cam (bottom and top) (C.4), needle segment holders (followers) (C.5), inner and outer converging cones (C.6, C.8), screws (C.7), outer cone opening (C.9) and needle segments (C.10). **(E)** The x-displacement of the followers over a 360° rotation of the cam, translating one stroke length (S) in the positive *x*-direction over 60° and one S in the negative *x*-direction over the following 300°. The graph shows linear (blue) and parabolic (red) displacement segments, ensuring a smooth cam path.

To achieve the 1:5 motion sequence, the needle segments had to be actuated simultaneously and continuously. Two actuation systems were designed: 1) one motorized unit for testing purposes (Figures 5A, B) and 2) one manual unit for the final prototype to minimize components and create a compact assembly (Figures 5C, D). A rotary cam-and-follower system was used for both actuators (Figures 5A, C, Part 4 in orange). The cams contained a groove, the cam path, guiding the followers (Figures 5A, C, Part 5 in green) to produce continuous linear translation (x-displacement) in the 1:5 motion sequence as the cam rotates. By attaching needle segments to the followers, the linear displacement was transferred to the needle segments, resulting in the 1:5 motion sequence of the biopsy needle overall.

To allow for sufficient space to clamp the needle segments into the followers, the needle segments transition from a larger diameter at the actuation unit to a smaller diameter at the needle tip. Similar to the design presented by Bloemberg et al. (2022), a double cone at the distal end of the actuation unit (Figure 5A, Part 8/Part 9 in blue/yellow; Figure 5C, Part 6/Part 8 in blue, yellow) guides the segments through S-shaped channels, to ensure smooth movement and prevent buckling. At the place where the needle segments diverge there is an opening in the outer cone (Figure 5A, Part 10; Figure 5C, Part 9) to allow the tissue core to exit the biopsy needle after transport.

For the motorized actuation unit, a regular barrel cam was used, where the followers surround the cam path. However, for the manual actuation unit, an inside-out cam design was chosen, with the cam path surrounding the followers. Furthermore, the cam used for manual actuation was star-shaped and approximately the size of a human palm [about 80 mm (Fallahi and Jadidian, 2011)] to allow for sufficient grip and thus easy manual actuation. Both cams shared the same cam path and thus the same stroke distance, resulting in the identical motion sequence of the followers in the longitudinal direction (Figure 5E), and were designed following guidelines from the Machinery’s Handbook [30th Edition, (Oberg et al., 2016)].

### 2.3 Prototype

**FIGURE 6:**
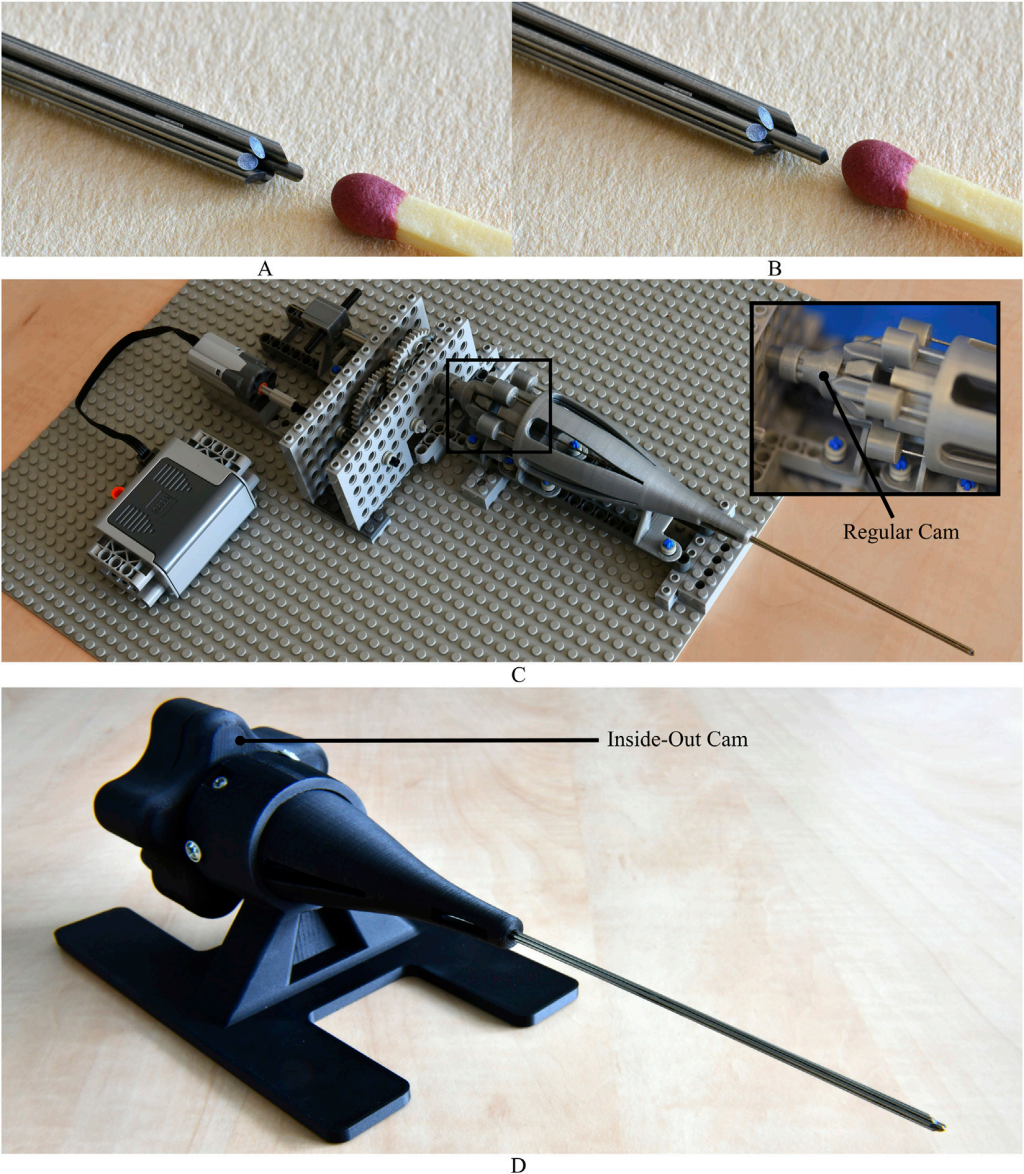
Prototype of the wasp-inspired biopsy needle. **(A)** Close-up of the needle tip, made of six nitinol rods interconnected using rings guided through slots in the needle segments. The stainless steel rings keep the segments in a hexagonal arrangement, resulting in a central lumen of 1 mm in diameter. **(B)** The lumen can be filled by a seventh needle segment. **(C)** Motorized actuation unit, which utilizes a regular (barrel) cam, created using 3D printing and off-the-shelf LEGO parts, to create the 1:5 motion sequence of the biopsy needle. **(D)** Manual actuator prototype, which utilizes an inside-out cam, fully produced using 3D printing, to create the 1:5 motion sequence of the biopsy needle.

The needle prototype consists of seven superelastic straightened nitinol rods, each 1 mm in diameter and 250 mm in length (Titaniumshop, Overijssel, the Netherlands). For the slot extrusion, Wire Electrical Discharge Machining (WEDM), known for its non-contact material removal process, was employed. This method allows for precise slot creation without imposing cutting forces on the segments (Prasad and Chakraborty, 2018; Charles et al., 2024). WEDM was used to create two L-shaped slots (10.25 mm × 0.40 mm) in the distal end of five needle segments. Additionally, a sixth needle segment, i.e., the carrier needle segment, featured smaller L-shaped slots (4.25 mm × 0.40 mm) to carry the rings forward. The length of these slots determines the maximum possible stroke length, which is equal to 4 mm. The seventh central needle segment can simply be inserted and removed easily when necessary. An additional 150 mm of length was added to the central needle segment for this purpose. Furthermore, WEDM was utilized to create the beveled tips of the needle segments (40°). The two interconnecting rings (outer diameter 2.1 mm, inner diameter 1.9 mm, length 2.0 mm) were made of stainless steel. Figure 6A shows the assembled needle excluding the central needle segment and Figure 6B shows the assembled needle including the central needle segment.

**FIGURE 7:**
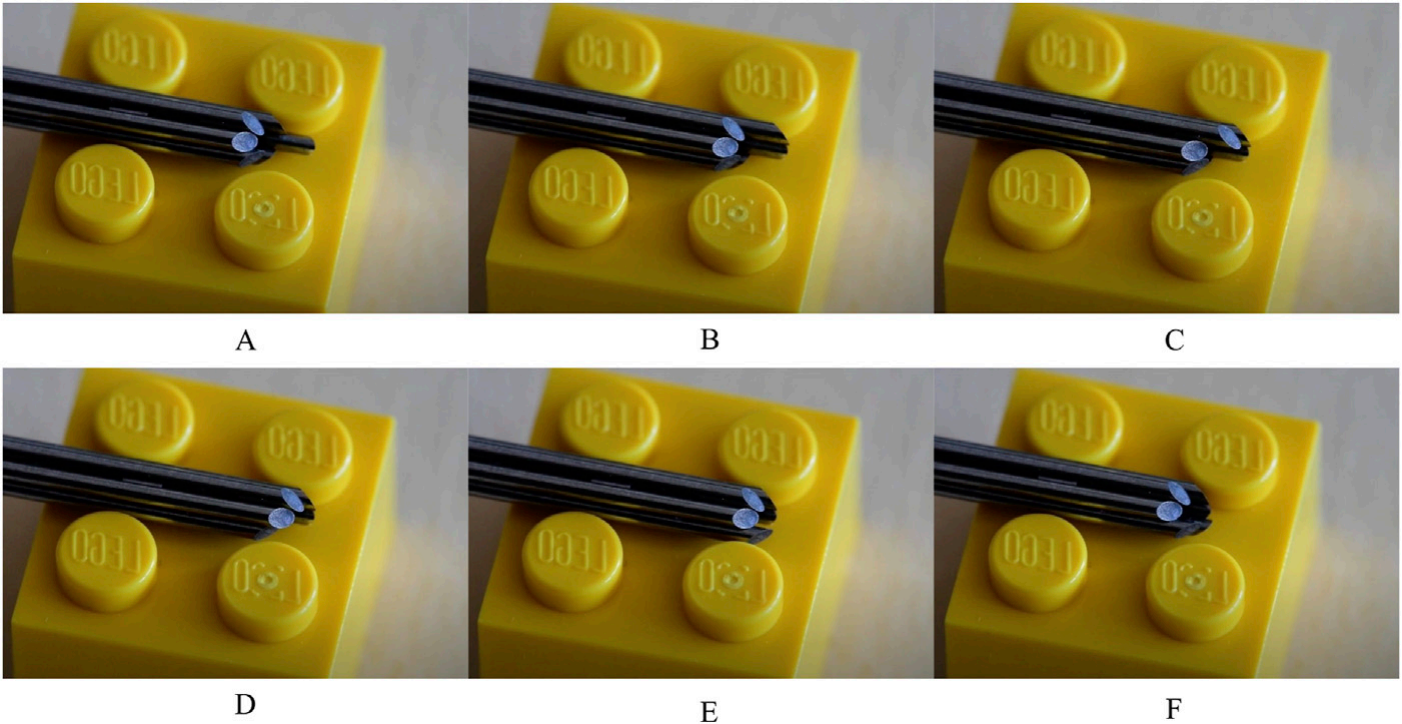
One cycle of needle segment translations without the central needle segment. Each 60-degree rotation of the cam results in the advancement of one needle segment over the stroke distance, while retracting the other five segments over one-fifth of this stroke distance. **(A–F)** correspond to the rotation of the cam over 60°, 120°, 180°, 240°, 300° and 360°, respectively.

The motorized actuation unit integrated off-the-shelf LEGO Technic parts and 3D-printed parts using Silver Metallic PLA on the Fused Deposition Modeling (FDM) printer Ultimaker S5 (Ultimaker, Utrecht, the Netherlands). A LEGO Technic Power Functions Medium motor with a rotational speed of 405 RPM (9V) in a no-load situation was used. A gearbox, created with LEGO gears, was used to adjust the rotational speed of the motor to the desired rotational speeds of the cam for the experiments. The manual actuation unit was entirely 3D printed out of Onyx (Markforged, Waltham, MA, United States), using the FDM printer Markforged Mark Two (Markforged, Waltham, MA, United States). For both actuators, the needle segments were glued into the followers using Loctite 401 Instant Adhesive (Loctite, Westlake OH, United States). Figure 6C and Figure 6D show the assemblies of the motorized and manual actuation units, respectively. Figure 7 illustrates the 1:5 motion sequence of the needle segments during actuation over one cycle.

## 3 Proof-of-principle evaluation

### 3.1 Experimental goal

**FIGURE 8:**
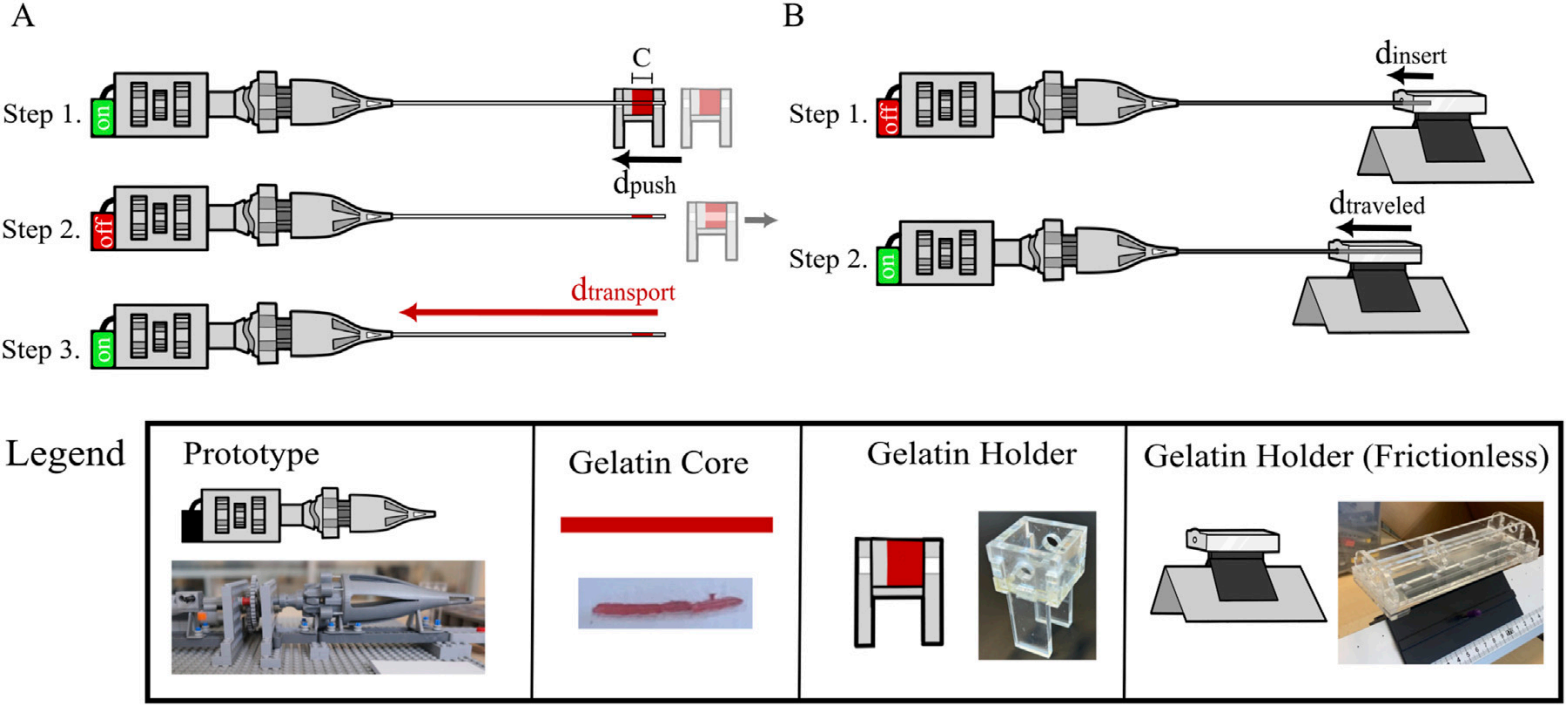
Schematic representation of Experiment 1 and 2 to test core-transport and self-propulsion principles. **(A)** Graphical overview of the experimental protocol of Experiment 1 “Core-Transport Capability”. Step 1: A gelatin block with width C in a gelatin holder is translated over the needle by a distance $d_{\text{push}}$ . Step 2: This leaves a gelatin core of length C in the needle. Step 3: The motor is turned on, causing the needle to transport the gelatin core over a distance $d_{\text{transport}}$ within $t_{\text{measured}}$ . **(B)** Graphical overview of the experimental protocol of Experiment 2 “Self-Propulsion Capability”. Step 1: A piece of gelatin in a frictionless gelatin holder is slid over the needle by a distance $d_{\text{insert}}$ with the motor off. Step 2: The motor is turned on, allowing self-propulsion to occur.

The goal of the evaluation of the wasp-inspired biopsy needle was twofold: 1) assessing the core-transport capability and 2) assessing the self-propulsion ability. These objectives were addressed in two separate experiments. Experiment 1 focused on core transport while Experiment 2 focused on self-propulsion. Figure 8 gives a schematic overview of both experiments. During testing, the setup involved moving tissue phantoms towards the prototype to enable keeping the prototype stationary. This contrasts a realistic scenario where the needle would advance through tissue, but since the relative movement between the needle and the tissue phantom is the same, it was assumed that this would not influence the test results. Data analysis and visualization were conducted using Python in PyCharm Community Edition 2024.1.1 (JetBrains, Prague, Czech Republic).

### 3.2 Experiment 1: core-transport capability

#### 3.2.1 Experimental variables

##### 3.2.1.1 Independent variables

###### 3.2.1.1.1 Core length

Core sample lengths typically range from 8 mm to 20 mm for pancreatic biopsies, with specific size requirements varying based on testing types, methods, and platforms (Gwoździewicz et al., 2023, Bhamidipati et al. (2021), Yap et al. (2016)). The capability of the prototype to transport tissue cores of different lengths (5 mm, 10 mm, 15 mm) was tested to validate its use for different core sizes.

###### 3.2.1.1.2 Stroke length

Larger stroke lengths theoretically lead to quicker core transport, as the core transport per cycle is dependent on the stroke length. To evaluate the effects of different stroke lengths on the efficiency and rate of transport, experiments were conducted using varying stroke lengths, up until the maximum allowable stroke length possible for the slot lengths of the prototype (2 mm, 3 mm, 4 mm).

###### 3.2.1.1.3 Rotational velocity

The rotational velocity of the cam determines the frequency of needle segment cycles per unit time. Higher rotational speeds could potentially accelerate tissue transport, optimizing biopsy procedure efficiency. Theoretical rotational speeds of 10.1 RPM, 24.3 RPM, and 50.6 RPM would be achieved using gearbox ratios of 0.025, 0.06, and 0.125, respectively.

###### 3.2.1.1.4 Tissue phantom elasticity

Gelatin was chosen as a tissue phantom due to its ability to mimic the mechanical properties of human tissue (Farrer et al., 2015; Amiri et al., 2022). Gelatin samples were prepared at different concentrations (5 wt%, 10 wt%, 15 wt%) by mixing porcine gelatin powder with boiled tap water, after which they were allowed to set for about 15 h in slab molds measuring 25 mm × 25 mm x 18 mm. The chosen concentrations correspond to moduli of elasticity of approximately 5.3 kPa, 17 kPa, and 31 kPa, respectively (Scali et al., 2019). This range was selected to approximate the mechanical properties of healthy pancreatic tissue, perilesional regions, and solid pancreatic tumors, with moduli of elasticity of 4 kPa, 23.9 kPa, and 42.9 kPa, respectively (Nabavizadeh et al., 2020).

##### 3.2.1.2 Dependent variables

###### 3.2.1.2.1 Transport efficiency

The transport efficiency (TE), measured in percentages, is crucial as it impacts procedural time and potentially the tissue sample quality. TE was quantified by comparing the measured core-transport duration $\left(t_{\text{measured}}\right)$ to the theoretical duration $\left(t_{\text{theoretical}}\right)$ over the transport distance $\left(d_{\text{transport}}\right)$ (Equation 5). The transport distance $\left(d_{\text{transport}}\right)$ , in mm, was calculated as the total needle length (120 mm), measured from the distal tip of the needle to the opening in the outer cone of the actuator, minus the core length. The measured transport time $\left(t_{\text{measured}}\right)$ in seconds, was visually assessed from video footage, where the starting time $\left(t_{\text{start}}\right)$ was the point at which the core was completely enclosed in the distal tip of the biopsy needle and the motor was turned on. The end time $\left(t_{\text{end}}\right)$ was the first moment the core could be seen entering the opening in the outer cone of the actuator. The theoretical transport time $\left(t_{\text{theoretical}}\right)$ , in seconds, was calculated by dividing the transport distance by the rotational speed of the cam (ω) , in RPM, which was assessed from the video footage, multiplied by the stroke length (S), in mm (Equation 6). The numerator was multiplied by a factor of 60 for conversion to seconds. Because we assume for our continuous mode of actuation that the tissue phantom core remains stationary with respect to the retracting needle segments, a factor of $\frac{6}{5}$ was used in the denominator.

$$TE=\frac{t_{\text{theoretical}}}{t_{\text{measured}}}\cdot 100\%$$ (5)

$$t_{\text{theoretical}}=\frac{d_{\text{transport}}\cdot 60}{\omega \cdot \frac{6}{5}S}$$ (6)

###### 3.2.1.2.2 Transport rate

To evaluate overall system performance, the transport rate (TR), measured in mm/s, was included as a dependent variable. It quantifies the biopsy core-transport distance within one second (Equation 7).

$$TR=\frac{d_{\text{transport}}}{t_{\text{measured}}}$$ (7)

#### 3.2.2 Experimental set-up

The experimental set-up included the motorized actuator assembly, held stationary, and a PolyMethyl MethAcrylate (PMMA) gelatin holder (with inner dimensions of 17 mm × 17 mm x 17 mm), which could translate linearly towards the prototype. To create the desired core length (C), the gelatin samples were cut to the appropriate size before use (17 mm × 17 mm x C mm). Unless indicated otherwise, a 0.06 gear ratio and 10 wt% gelatin were used.

#### 3.2.3 Experimental protocol

The core-transport assessment was split into two sub-experiments. Table 1 provides the experimental conditions for the different sub-experiments.

**TABLE 1:** Experimental conditions for Experiment 1A and 1B.

Condition	Stroke length [mm]	Core length [mm]	Gear ratio	Gelatin concentration [wt%]
Experiment 1A				
A1	2	5	0.06	10
A2	2	10	0.06	10
A3	2	15	0.06	10
A4	3	5	0.06	10
A5	3	10	0.06	10
A6	3	15	0.06	10
A7	4	5	0.06	10
A8	4	10	0.06	10
A9	4	15	0.06	10
Experiment 1B				
B1	from 1A*	from 1A*	0.025	10
B2	from 1A*	from 1A*	0.125	10
B3	from 1A*	from 1A*	0.06	5
B4	from 1A*	from 1A*	0.06	15

*The stroke length and core length that yielded the highest transport efficiency in Experiment 1A were used in Experiment 1B.

Experiment 1A evaluated the effect of the gelatin core length and stroke length on the transportation efficiency and rate in a 3 x 3 factorial design, exploring potential interaction effects between the parameters.

In Experiment 1B, the impact of the rotational velocity and gelatin elasticity on the transport efficiency and transport rate was assessed, using the core and stroke length combination that achieved the highest transport efficiency in Experiment 1A.

For both sub-experiments, the same procedure was followed. To create a transportable core, the needle self-propelled through the gelatin until the holder had translated 30 mm. During this translation, the biopsy needle cut out a core with a diameter of 1 mm $\left(\emptyset_{\text{inner}}\right)$ . After this translation, the motor was turned off and the gelatin holder was removed. The motor was then turned on again, leading to the transportation of the gelatin core from the distal tip of the biopsy needle to the opening in the outer cone of the actuator. Each experiment was recorded using a video camera focused on the outer cone opening. The experiment concluded when the core became visible in the opening. The core was then removed using tweezers, and the needle was cleaned with lukewarm water and tweezers to ensure the removal of gelatin debris from the inside. Each condition was repeated five times. Figure 8A. shows a schematic overview of this protocol.

#### 3.2.4 Experimental results

##### 3.2.4.1 Core length and stroke length

**FIGURE 9:**
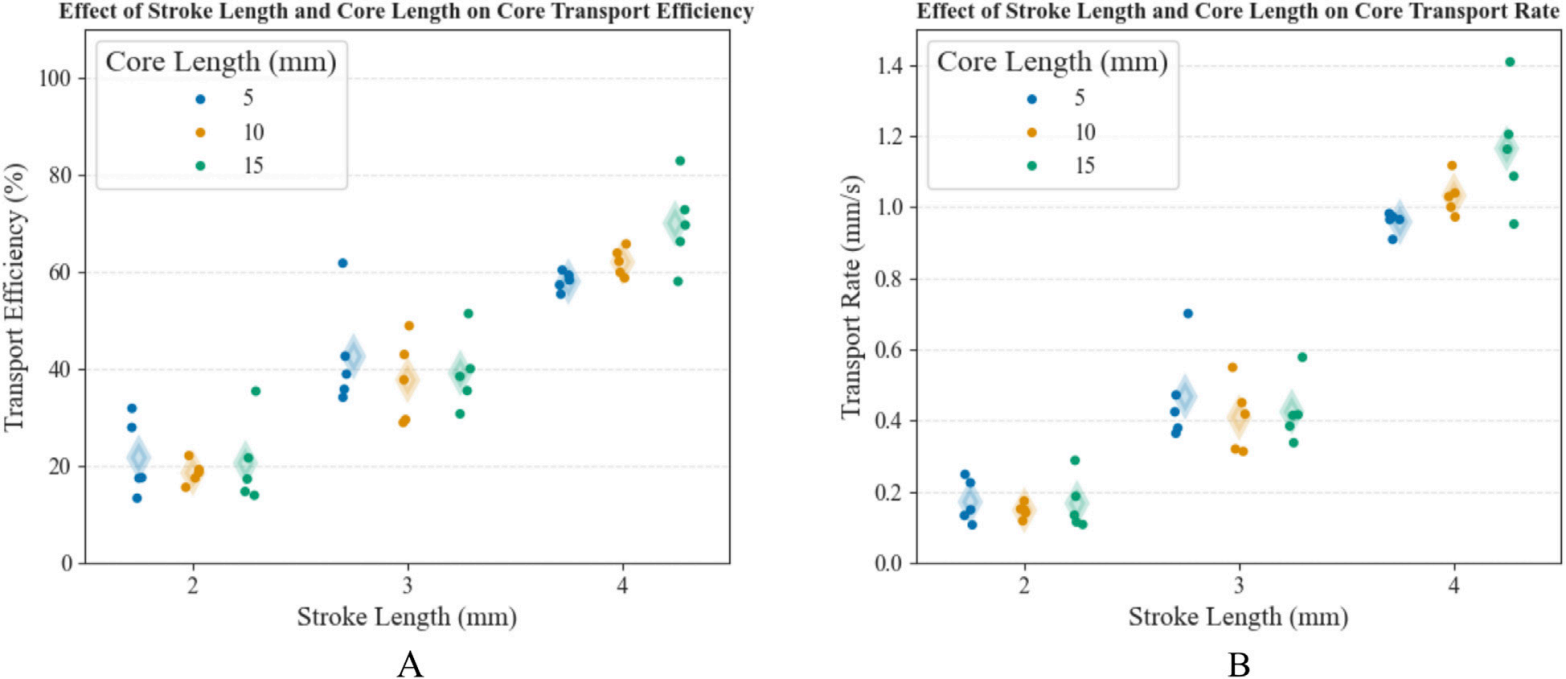
Grouped strip plots displaying **(A)** the transport efficiency (in %) and **(B)** the transport rate (in mm/s) for different test conditions; using stroke lengths of 2 mm, 3 mm, and 4 mm and core lengths of 5 mm, 10 mm, and 15 mm, respectively. The mean is indicated by a translucent diamond.

Table 2 shows the mean and Standard Deviation (SD) of the TE and TR per condition of Experiment 1A. The combination of a stroke length of 4 mm and a core length of 15 mm yielded the highest mean transport efficiency (69.9% ± 9.1%) and transport rate (1.16 mm/s ± 0.17 mm/s). These parameters were, therefore, used in Experiment 1B. The lowest mean transport efficiency (18.6% ± 2.4%) and mean transport rate (0.15 mm/s ± 0.02 mm/s) was achieved by the combination of a 2-mm stroke length and 10-mm core length. The results of Experiment 1A are visualized in separate grouped strip plots (Figure 9).

**TABLE 2:** Test results of Experiment 1A, including the conditions (S, stroke distance [mm], C, core length [mm]), and the resulting mean and Standard Deviation (SD) of the Transport Efficiency (TE) and the Transport Rate (TR). Each condition was tested five-fold. The maximum and minimum TE and TR are indicated in bold.

Condition	TE [%]	TR [mm/s]
A1 (S2-C05)	21.6 ± 7.9	0.17 ± 0.06
A2 (S2-C10)	**18.6** ± **2.4**	**0.15** ± **0.02**
A3 (S2-C15)	20.6 ± 8.8	0.17 ± 0.07
A4 (S3-C05)	42.7 ± 11.2	0.47 ± 0.14
A5 (S3-C10)	37.6 ± 8.6	0.41 ± 0.10
A6 (S3-C15)	38.2 ± 7.7	0.43 ± 0.09
A7 (S4-C05)	58.1 ± 1.9	0.96 ± 0.03
A8 (S4-C10)	62.1 ± 2.9	1.03 ± 0.05
A9 (S4-C15)	**69.9** ± **9.1**	**1.16** ± **0.17**

##### 3.2.4.2 Rotational velocity and gelatin elasticity

**FIGURE 10:**
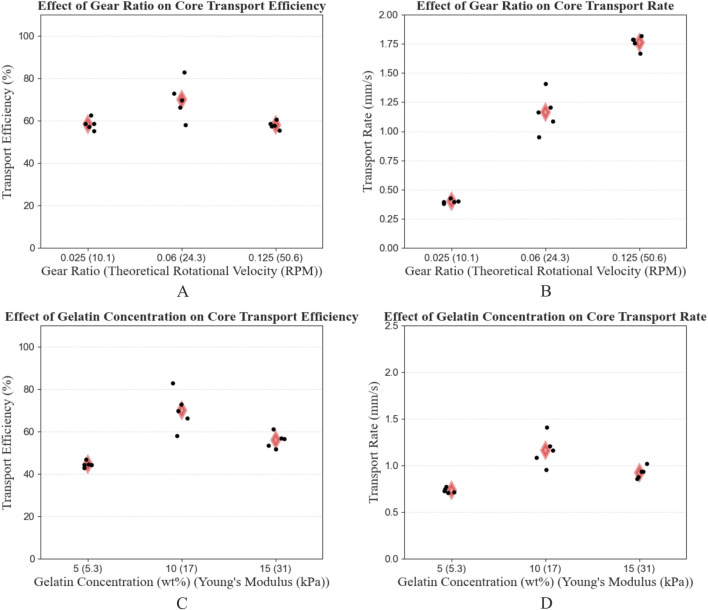
Strip plots showing the Transport Efficiency (TE) and Transport Rate (TR) for different gear ratios **(A, B)** and gelatin concentrations **(C, D)**. The mean per conditional group is indicated by a red diamond. The results from the 0.06 (24.3 RPM) gear ratio and 10 wt % (17 kPa) gelatin concentration condition are taken from Experiment 1A.

Table 3 shows the mean and SD of the TE and TR per condition of Experiment 1B. Additionally, the measured angular velocity (ω) of the cam is noted, as this differs from the theoretical value. The 0.06 gear ratio with 10 wt% gelatin concentration group presented the highest mean transport efficiency (69.9% ± 9.1%) and transport rate (1.16 mm/s ± 0.17 mm/s). The lowest mean transport efficiency (44.6% ± 1.4%) was achieved by the combination of a gear ratio of 0.06 and a gelatin concentration of 5 wt%. The lowest mean transport rate (0.40 mm/s ± 0.02 mm/s) was achieved by the combination of a gear ratio of 0.025 and a gelatin concentration of 10 wt%. The results of Experiment 1B are visualized in separate strip plots (Figure 10).

**TABLE 3:** Test results of Experiment 1B, showing the conditions (S = stroke distance [mm], C = core length [mm], GR = gear ratio, GC = gelatin concentration [wt%]), as well as the mean and standard deviation (SD) of the angular velocity of the cam (ω) , the Transport Efficiency (TE), and the Transport Rate (TR).

Condition	ω [RPM]	TE [%]	TR [mm/s]
A9 (S4 - C15)*	20.8 ± 0.3	**69.9** ± **9.1**	**1.16** ± **0.17**
B1 (GR025 - GC10)	8.5 ± 0.07	58.4 ± 2.8	**0.40** ± **0.02**
B2 (GR125 - GC10)	38.0 ± 0.9	58.0 ± 1.8	1.76 ± 0.06
B3 (GR060 - GC05)	20.6 ± 0.3	**44.6** ± **1.4**	0.73 ± 0.03
B4 (GR060 - GC15)	20.7 ± 0.1	55.9 ± 3.6	0.92 ± 0.06

*These results are taken from Experiment 1A. The maximum and minimum TE and TR are indicated in bold.

### 3.3 Experiment 2: self-propulsion capability

#### 3.3.1 Experimental variables

##### 3.3.1.1 Independent variables

The experiment did not have any independent variables.

##### 3.3.1.2 Dependent variables

###### 3.3.1.2.1 Self-propelling rate

The self-Propelling Rate (PR), measured in mm/s, directly affects the procedural duration. It is defined as the distance $\left(d_{\text{travel}}\right)$ , in mm, the needle self-propels within a set time $\left(t_{\text{travel}}\right)$ , in seconds (Equation 8). $t_{\text{travel}}$ was fixed at 120 s, and millimeter paper was used to measure the self-propelled distance.

$$PR=\frac{d_{\text{travel}}}{t_{\text{travel}}}$$ (8)

###### 3.3.1.2.2 Self-propelling efficiency

The efficiency of self-propulsion was assessed by the Slip Ratio (SR). The SR quantifies the relative slip of the needle as it propels. We assumed that slip was the sole reason that the measured self-propelling rate $\left(PR_{\text{measured}}\right)$ was lower than the theoretical self-propelling rate $\left(PR_{\text{theoretical}}\right)$ . The SR is calculated using the $PR_{\text{measured}}$ and $PR_{\text{theoretical}}$ in mm/s (Equation 9). The measured self-propelling rate was determined experimentally by assessing how far the needle could self-propel within a given time. The theoretical self-propelling rate was derived from the rotational velocity of the cam $\left(\omega_{\text{actual}}\right)$ in RPM, measured from video footage and divided by 60 to get the rotations per second, and the stroke length in mm, multiplied by a factor of $\frac{6}{5}$ to account for the continuous actuation of the needle segments where we assume for the self-propelling motion that the retracting needle segments remain stationary with respect to the tissue phantom, while the advanced needle segment moves forward (Equation 10).

$$SR=1-\frac{PR_{\text{measured}}}{PR_{\text{theoretical}}}$$ (9)

$$PR_{\text{theoretical}}=\frac{\omega_{\text{actual}}}{60}\cdot \frac{6}{5}S$$ (10)

#### 3.3.2 Experimental set-up

The motorized actuator prototype was aligned with a PMMA gelatin holder (with inner dimensions of 80 mm × 20 mm x 20 mm). Gelatin of 10 wt% was used after it had been allowed to set for about 15 h. The gelatin holder fit on an air track (Eurofysica), which facilitated near-frictionless linear translation of the gelatin holder towards the actuation unit. The central needle segment filled the lumen during testing. The gelatin holder was translated manually over a distance of 20 mm $\left(d_{\text{insert}}\right)$ . This manual insertion was done to ensure there was sufficient initial contact between the needle surface and the gelatin to facilitate the friction-based self-propulsion. The stroke length used was 4 mm and the gear ratio was 0.06, yielding a theoretical rotational speed of 24.3 RPM.

#### 3.3.3 Experimental protocol

Figure 8B illustrates the protocol, which was repeated five times. After manual insertion, the motor was turned on, allowing the gelatin to translate toward the prototype. After 120 s, the motor was turned off and the exact traveled distance was determined from the video footage. Finally, the needle was cleaned with lukewarm water to remove potential gelatin debris.

#### 3.3.4 Experimental results

Within the experimental group, the mean PR and SR, including SD, were, respectively, PR = 0.247 ± 0.061 [mm/s] and SR = 0.842 ± 0.042.

### 3.4 Proof-of-principle of sequential functioning

As a proof of principle, it was assessed whether the self-propulsion and core transport could be performed sequentially. Using the set-up of Experiment 2, the wasp-inspired biopsy needle was inserted 20 mm into a gelatin block (10 wt%), measuring 80 mm in total length. The motor was turned on, allowing the needle to self-propel over a distance of 45 mm, after which the central needle segment was removed by pulling it out to allow for core extraction over the remaining 15 mm, yielding a core of 15 mm. The near-frictionless air track was then deactivated, immobilizing the gelatin block as the gelatin core was transported from the distal tip to the opening in the outer cone of the actuator. A video showing the proof-of-principle of sequential functioning can be found in the supplementary material.

## 4 Discussion

### 4.1 Main findings

The proof-of-principle evaluation implied two main findings. It showed that the wasp-inspired biopsy needle was: 1) able to transport a gelatin core, mimicking tissue, using friction-based transport and 2) was able to self-propel. Additionally, it showed that self-propulsion and core transport could be performed sequentially.

The optimal conditions for core transport in our needle design included a 4-mm stroke length, 15-mm core length, theoretical rotational velocity of 24.3 RPM (actual 20.8 RPM), and 10-wt% gelatin composition, achieving a transport efficiency of 69.9% ± 9.1% and a transport rate of 1.16 mm/s ± 0.17 mm/s. Increasing the stroke length not only enhanced the transport rate as expected but also improved efficiency, resulting in a positive impact on the overall transport rate. This phenomenon might be attributed to the advancing needle segment having a higher velocity as the stroke length is increased. A higher velocity could lead to relatively lower friction forces between the advancing needle segment and the gelatin core, leading to more efficient core transport. The flexible wasp-inspired tissue transport design of de Kater et al. (2021) was able to transport tissue with a similar transport rate of 0.83 ± 0.08 mm/s under similar circumstances (horizontal orientation, 10-wt% gelatin, 25 RPM, stroke length of 5.2 mm), despite the larger lumen (3.8 mm). During current CB procedures, tissue cores measuring 1–2 cm in length are typically obtained (Sabel, 2009), which is similar to the core lengths retrieved during our core-transport assessment. Moreover, our needle design can theoretically transport tissue cores up to the entire length of the needle, which is challenging for aspiration-based devices due to clogging issues.

The self-propulsion capability evaluation showed the needle achieving a slip ratio of 0.842 ± 0.042 and a self-propelling rate of 0.257 ± 0.0611 mm/s. In comparison, the self-propelling needle by Scali et al. (2019) (diameter 0.8 mm), also using 10-wt% gelatin, exhibited a lower mean slip ratio of 0.3. The larger diameter of our prototype ( $\emptyset_{\text{outer}}$ : 3 mm) may contribute to increased gelatin displacement and rupturing around the needle, leading to the formation of a cavity around the needle, thereby reducing the contact area between the needle and surrounding gelatin. A reduction in the contact area between the needle segments and the gelatin will reduce the overall friction force, making it less likely that the friction force of the retracting needle segments can counterbalance the cutting and friction force on the advancing needle segment, causing slip. Miniaturizing the outer dimensions of the needle could potentially mitigate this phenomenon.

The wasp-inspired biopsy needle offers significant advantages over conventional biopsy needles for percutaneous biopsy procedures. It avoids buckling and clogging due to its friction-based propulsion and core-transport methods, allowing for downsizing while maintaining the capability to transport intact tissue samples for histological examination.

### 4.2 Limitations and future research

During the evaluation, gelatin was chosen as a suitable tissue phantom due to its elasticity that can resemble human tissue, cost-effectiveness, and accessibility. However, gelatin phantoms do not fully capture the complexity of real tissues, particularly concerning the heterogeneity of human tissue, in specific tumor tissue (Polyak, 2011). Due to its homogeneity, gelatin exhibits brittle fracture behavior, leading to sudden rupturing when subjected to mechanical stress (Czerner et al., 2013). This could lead to increased fragmentation during core transport and could lead to cavity formation around the needle during self-propulsion. Furthermore, for needle propulsion experiments, other mechanical properties than the elasticity, for example, the friction coefficient between needle and tissue and the tissue’s shear modulus and ultimate strength, could also be of interest. Transitioning to human tissue poses challenges such as variable stiffness between tissue types (Okamura et al., 2004), complicating self-propulsion. For instance, Bloemberg et al. (2022) reported slip ratios ranging from 0.86 to 0.96 in *ex vivo* prostate tissue. An interesting next step would therefore be to validate the wasp-inspired biopsy needle in complex tissue models like multi-layered phantoms and *ex vivo* tissue. When moving towards *ex-vivo* tissue studies and clinical trials, developing protocols in compliance with the ISO 13485 standard and the Medical Device Regulation 2017/745 will enable us to compare our needle with commercially available alternatives.

The self-propulsion principle of the wasp ovipositor that was implemented in our needle design allowed the needle to self-propel through different gelatin substrates. In order for the self-propulsion principle of the needle to hold, continuous contact between the outer needle surface and the substrate is required. Therefore, during our self-propulsion evaluation, the needle was manually inserted by 20 mm in the gelatin phantom before being actuated. In clinical practice, the needle first has to puncture the skin of the patient, before being able to self-propel through the tissue. The skin introduces a surface stiffness force due to the needle puncturing the skin until the moment of puncture (Okamura et al., 2004). To overcome this, manual insertion of the needle through the skin could be an option using an initial puncture needle, ensuring sufficient contact between the outer needle surface and the surrounding tissue.

The current 3-mm outer diameter of the wasp-inspired biopsy needle prototype represents an improvement over larger former tissue-transport designs [( $\emptyset_{\text{outer}}$ : 7 mm, $\emptyset_{\text{outer}}$ : 10 mm) (Sakes et al., 2020; de Kater et al., 2021)] for the use in biopsy procedures, but falls short of standard biopsy needles (18 G, outer diameter of 1.27 mm). Future efforts should therefore focus on miniaturization the needle. The current slot manufacturing method could allow for prototype production on a smaller scale using nitinol rods and stainless steel capillary tubes with smaller diameters. However, further miniaturization may be limited by WEDM, typically using wires of 0.15–0.3 mm (Charles et al., 2024). However, high precision WEDM, capable of handling wires as fine as 0.025–0.1 mm (Slǎtineanu et al., 2020) could, in theory, produce smaller slots and, therefore, aid in further miniaturizing the needle dimensions.

Additionally, given the hexagonal arrangement, reducing the outer diameter will also decrease the lumen diameter, thereby decreasing the amount of tissue that can be retrieved. Since the required amount of tissue differs per diagnostic test (Bhamidipati et al., 2021), to enhance functionality, a relatively large lumen is preferred. To increase the lumen diameter, we propose employing multiple central needle segments or a single central needle segment with a larger diameter.

Navigating around vital structures during percutaneous biopsy procedures, especially near the pancreas where structures like the bowel, liver, kidney, or major vessels can block direct access (Lin et al., 2017), is challenging. Incorporating steerability into biopsy needles can aid in overcoming this challenge. Bevel tips, commonly used in intravascular injections (Smuck et al., 2010), prove effective for maneuvering within the body. Research by Scali et al. (2019) shows successful needle steering using six nitinol rods by simulating an approximated bevel-shaped tip. Alternatively, a prebend central needle segment could be employed to facilitate needle steering. Incorporating either approach into our biopsy needle design could further enhance its functionality.

Our current needle design showcases two use scenarios: (1) needle propulsion, and (2) substrate transportation from the needle tip to the needle base. On top of that, the functionality of our biopsy needle could inspire designs beyond its scope as the motion sequence could also be reversed to facilitate the delivery of substances from the needle base to the needle tip, such as the delivery of medicine, radioactive particles, or high-viscosity hydrogels for cartilage repair, to enhance the delivery of low-viscosity samples compared to current expulsion-based delivery methods (Øvrebø et al., 2022).

## 5 Conclusion

In conclusion, our study introduces a wasp-inspired biopsy needle capable of both self-propulsion and friction-based core transport. Drawing from previous wasp-inspired designs, we integrated these functionalities by transitioning from external interconnecting structures to a ring passing through slots along the needle segments. The prototype demonstrated the ability to perform these functionalities separately and sequentially, offering a viable alternative to current biopsy needles that are prone to buckling and clogging during downsizing. This innovation is particularly promising for procedures requiring long, thin, and precise biopsy needles, such as in percutaneous pancreatic biopsies. Further advancements, including miniaturization and the integration of steering could expand the biopsy needle’s application scope, potentially improving tissue sampling accuracy and reducing patient complications.

## Data Availability

The datasets presented in this study can be found in online repositories. The data is available via doi:10.4121/72a188cc-0d15-41b5-b422-9d1650f6fcf3.
